# Docosahexaenoic Acid Coordinating with Sodium Selenite Promotes Paraptosis in Colorectal Cancer Cells by Disrupting the Redox Homeostasis and Activating the MAPK Pathway

**DOI:** 10.3390/nu16111737

**Published:** 2024-06-01

**Authors:** Sheng Zhao, Yuzhou Meng, Wenxun Cai, Qiwen Luo, Hongyang Gao, Qiang Shen, Dongyun Shi

**Affiliations:** 1 Key Laboratory of Metabolism and Molecular Medicine of the Ministry of Education, Department of Biochemistry and Molecular Biology, School of Basic Medical Sciences, Fudan University, Shanghai 200032, China; 2 Institute of Electronmicroscopy, School of Basic Medical Sciences, Fudan University, Shanghai 200032, China; 3 Free Radical Regulation and Application Research Center of Fudan University, Shanghai 200032, China

**Keywords:** colorectal cancer, docosahexaenoic acid, sodium selenite, synergistic anticancer effects, reactive oxygen species, cell paraptosis, redox state, MAPKs

## Abstract

Tumor cells are characterized by a delicate balance between elevated oxidative stress and enhanced antioxidant capacity. This intricate equilibrium, maintained within a threshold known as redox homeostasis, offers a unique perspective for cancer treatment by modulating reactive oxygen species (ROS) levels beyond cellular tolerability, thereby disrupting this balance. However, currently used chemotherapy drugs require larger doses to increase ROS levels beyond the redox homeostasis threshold, which may cause serious side effects. How to disrupt redox homeostasis in cancer cells more effectively remains a challenge. In this study, we found that sodium selenite and docosahexaenoic acid (DHA), a polyunsaturated fatty acid extracted from marine fish, synergistically induced cytotoxic effects in colorectal cancer (CRC) cells. Physiological doses of DHA simultaneously upregulated oxidation and antioxidant levels within the threshold range without affecting cell viability. However, it rendered the cells more susceptible to reaching the upper limit of the threshold of redox homeostasis, facilitating the elevation of ROS levels beyond the threshold by combining with low doses of sodium selenite, thereby disrupting redox homeostasis and inducing MAPK-mediated paraptosis. This study highlights the synergistic anticancer effects of sodium selenite and DHA, which induce paraptosis by disrupting redox homeostasis in tumor cells. These findings offer a novel strategy for more targeted and less toxic cancer therapies for colorectal cancer treatment.

## 1. Introduction

Colorectal cancer (CRC), which ranks as the third most prevalent malignancy globally, represents a significant threat to public health and overall well-being. The aggressive behavior, poor prognosis, and lack of targeted therapy for CRC pose formidable challenges to its treatment [1]. Surgery remains the primary treatment option for CRC, while adjuvant chemotherapy has also been widely used to improve the survival outcomes of patients with stage II or higher CRC [2,3]. Nevertheless, the adverse effects of chemotherapy limit the effectiveness of adjuvant chemotherapy and seriously affect patient prognoses [4,5]. There is an urgent necessity to revolutionize adjuvant chemotherapy regimens and explore alternative treatment modalities to tackle this problem.

Selenium is an essential trace element for human health and the immune system [6]. As a constituent of selenoproteins, selenium plays a crucial role in antioxidant systems, particularly within the glutathione peroxidase family (GPXs), where it contributes to scavenging reactive oxygen species (ROS) and protecting cells from oxidative stress [7]. Previous studies have revealed that selenium exhibits both antioxidant and pro-oxidative stress effects, with the effects depending on dosage and selenium form [8]. High concentrations of sodium selenite have shown promising therapeutic potential in ovarian cancer, breast cancer, and thyroid cancer [9,10,11,12]. It exerts its therapeutic effects by inducing ROS and triggering multiple cell death machineries, including apoptosis and ferroptosis, in cancer cells. Two clinical studies utilizing high-dose sodium selenite in patients with advanced tumors have achieved favorable outcomes [13,14]. Although selenite salts exhibit specific cytotoxicity towards cancer cells, we cannot ignore the adverse effects induced by high doses of sodium selenite, such as abdominal pain, alopecia, nail changes, neurological symptoms, and even organ damage [15]. Dosage reduction remains a critical issue to be solved.

In recent years, there has been a growing interest in utilizing key compounds derived from natural sources for therapeutic purposes [16]. Docosahexaenoic acid (DHA) is a long-chain Omega-3 fatty acid that can be obtained from marine organisms [17]. DHA functions as a potent antioxidant and plays a crucial role in neural tissues such as the brain and retina [18]. Multiple clinical studies have demonstrated that oral supplementation of DHA can significantly improve the oxidative stress state in the blood [19], alleviate age-related ocular diseases [20], and provide various benefits, including neuroprotection [21]. Additionally, its potential role in cancer therapy is increasingly gaining attention. Supplementation with DHA is deemed advantageous for meeting the nutritional needs of cancer patients, thereby decreasing the incidence of cachexia [22]. As such, a multitude of preclinical studies have demonstrated that DHA supplementation can increase the abundance of polyunsaturated fatty acids (PUFAs) in cell membranes, thereby promoting lipid peroxidation and ferroptosis and ultimately exerting pro-oxidative effects [23,24,25,26]. However, the mechanism by which this antioxidant exerts pro-oxidative effects remains unclear. 

Tumor cells exhibit an abnormal redox state with high oxidative–reductive equilibrium [27], making them sensitive to redox regulation [28]. Meanwhile, DHA and sodium selenite, both of which are essential for health, exert regulatory effects on ROS. This study investigates the potential of a combination therapy using DHA and sodium selenite (SDCT) to enhance therapeutic effects while minimizing toxicity for effective CRC treatment. This research focuses on regulating the redox homeostasis of tumor cells, evaluating therapeutic efficacy, and exploring the underlying mechanisms of action.

## 2. Materials and Methods

### 2.1. Cell Lines and Cultures 

The human CRC cell line SW620 (Rosetta Stone Biotechnology, Jinan, China) was cultured in RPMI 1640 medium (Gibco, Carlsbad, CA, USA). RKO cells (Meilunbio, Dalian, China) were maintained in MEM medium (MA0217, Meilunbio). Both media were supplemented with 10% fetal bovine serum (FBS) (Yeasen, Shanghai, China) and incubated at 37 °C in a humidified atmosphere containing 5% CO_2_. All cultures were supplemented with 1× streptomycin and penicillin.

### 2.2. Reagents and Antibodies

DHA (D100925), purchased from Aladdin (Shanghai, China), was dissolved in ethanol [24]. N-acetylcysteine (NAC, N170063) and cycloheximide (CHX, C112766) were purchased from Aladdin (CHN). Sodium selenite (S5121) was purchased from Sigma (St. Louis, MO, USA).

The following antibodies were utilized in this study: ERK1/2 (ET1601-29) and Phospho- ERK1 (T202) + ERK2 (T185) (ET1603-22) were purchased from HUABIO (Hangzhou, China). The following antibodies were purchased from ABclonal (Wuhan, China): Phospho-p38 (AP1508), p38 (A14401), HO-1 (A1346), and GPX4 (A21440). The following antibodies were purchased from Proteintech (Wuhan, China): Nrf2 (16396-1-AP), AMPK α2 (18167-1-AP), β-Tubulin (11224-1-AP), and β-actin (20536-1-AP). Phospho-AMPKα1/α2 (11183) antibodies were procured from Signalway Antibody (College Park, MD, USA). The secondary antibodies, anti-mouse-IgG-HRP and anti-rabbit-IgG-HRP, were purchased from Signalway Antibody.

### 2.3. Cell Proliferation

The cell proliferation assay was performed using the CCK-8 (Cell Counting Kit-8, MA0218, Meilunbio, China) assay, following the manufacturer’s instructions. Cells were seeded at a density of 2 × 10^4^ cells per well in 96-well plates, incubated for 24 h, and then treated with sodium selenite (0, 5.78, 11.56, 17.34, 23.12, or 28.9 µM) or DHA (0, 6.25, 12.5, 25, or 50 µM) for 24 h. After treatment, a CCK-8 solution was added and incubated for 0.5–4 h. The absorbance was then measured at a wavelength of 450 nm using a microplate reader.

### 2.4. Live/Dead Staining Assay

SW620 cells were seeded at a density of 2 × 10^4^ cells per well in 96-well plates, incubated for 24 h, treated with DHA or sodium selenite for 24 h, and stained using a Calcein-AM/PI staining kit (Beyotime, Shanghai, China) at 37 °C for 15 min. PI stained the dead cells, while Calcein AM stained the live cells. The samples were imaged using a fluorescent microscope.

### 2.5. MDA Assay

SW620 cells were seeded at a density of 4 × 10^5^ cells per well in 6-well plates, incubated for 24 h, and then treated with sodium selenite or DHA for 24 h. Then, MDA was performed according to a previously described protocol [29]. Briefly, MDA was quantified by the reaction of thiobarbituric acid (TBA) with MDA, forming a stable fluorescent derivate. The fluorescent intensity was determined with a wavelength of Ex = 515 nm and Ep = 553 nm by a microplate reader.

### 2.6. Intracellular ROS Assay

The fluorescent probe 2′,7′-dichlorodihydrofluorescein diacetate (H_2_DCFDA) was employed to measure the intercellular ROS of SW620 cells, which were seeded into a 6-well culture plate at a density of 4 × 10^5^ cells per well for 24 h and then treated with sodium selenite or DHA for 12 h after that. The cells were collected, incubated with 10 µM H_2_DCFDA for 30 min, and washed with PBS once. The samples were imaged using a fluorescent microscope, and the fluorescent intensity was determined by a NovoCyte Quanteon flow cytometer.

### 2.7. GSH and GSSG Assay

SW620 cells were seeded at a density of 4 × 10^5^ cells per well in 6-well plates, incubated for 24 h, and then treated with sodium selenite or DHA for 24 h. Then, GSH and GSSG were tested according to Kand’ár R’s described protocol [30]. Briefly, GSH reacts with orthophthaldehyde (OPA) to form a stable, highly fluorescent derivate at pH 8. While at the measurement of GSSG, GSH was complexed with N-ethylmaleimide (NEM), and the fluorescent derivate was formed at pH = 12. The fluorescence intensity was determined with a wavelength of Ex = 340 nm and Ep = 420 nm by a microplate reader. 

### 2.8. Western Blotting Assay

SW620 cells were seeded at a density of 4 × 10^5^ cells per well in 6-well plates, incubated for 24 h, and then treated with sodium selenite or DHA for 24 h. After that, total proteins from the treated cells were extracted by RIPA (Beyotime) buffer containing a 1 mmol/L cocktail (Yeasen). Protein bands were separated by SDS–PAGE (5% concentrating gel and 10–15% separation gel, depending on the molecular weight of the target protein) and then transferred to a 0.45 μm polyvinylidene difluoride (PVDF) membrane (Millipore Corp., Bedford, MA, USA). After blocking with 5% non-fat milk, the membranes were respectively probed with primary antibodies and HRP-conjugated secondary antibodies. Protein expression was then visualized on the Tanon-5200 chemiluminescent imaging system (Tanon Science Technology, Shanghai, China). The quantification of the indicated protein bands was assessed using ImageJ version 1.53 software.

### 2.9. Transmission Electron Microscopy (TEM)

SW620 cells were seeded at a density of 4 × 10^5^ cells per well in 6-well plates, incubated for 24 h, and then treated with sodium selenite or DHA for 12 h. The samples were examined with a Jeol Jem-100SV electron microscope (Tokyo, Japan), which was operated at 80 Kv after being fixed by 3% glutaraldehyde in 0.1 M phosphate buffer (pH 7.3) at the Institute of Electron microscopy, Shanghai Medical College of Fudan University.

### 2.10. Statistical Analysis

Experimental data were obtained by at least three independent experiments and analyzed using GraphPad Prism 9.0 software. The two-tailed unpaired Student’s *t*-test and one-way analysis of variance (ANOVA) were employed to identify if there were significant differences between the data of different groups. Data were considered statistically significant when the *p*-value was less than 0.05.

## 3. Results

### 3.1. The SDCT Could Induce Cell Death in CRC Cells

**Figure 1:**
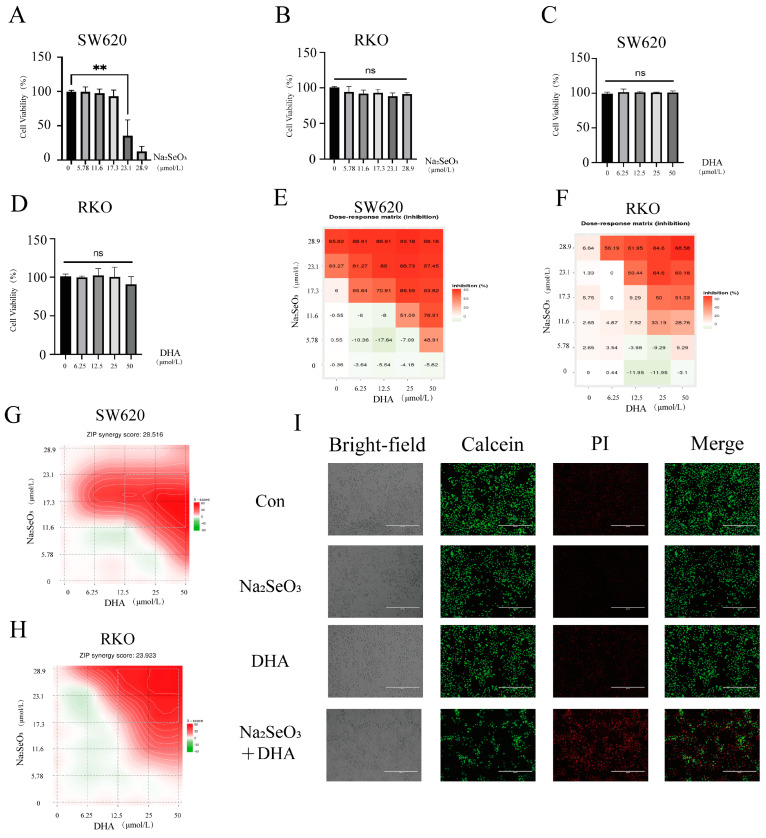
The SDCT could induce cell death in CRC cells. (**A**,**B**) SW620 and RKO cells were exposed to sodium selenite for 24 h and then the cell viability was determined by the CCK8 assay, as described in the Section 2. (**C**,**D**) SW620 and RKO cells were exposed to DHA for 24 h, and then the cell viability was determined by the CCK8 assay. (**E**–**H**) SW620 and RKO cells were exposed to sodium selenite and DHA for 24 h, and then the cell viability was determined by the CCK8 assay. The dose–response matrix and synergy score were determined by SynergyFinder 2.0. (**I**) SW620 cells were exposed to 11.5 µM sodium selenite and 25 µM DHA for 24 h and subjected to Calcein-AM/PI staining (Calcein AM: live cells; PI: dead cells). Scale bar: 400 µm. The representative images are shown. The corresponding quantitative histograms from three independent experiments were shown (The values represent the mean ± SD, n = 3. Two-tailed unpaired Student’s *t*-test. ns: not significant; ** *p* < 0.01).

Sodium selenite has shown promising effects on inhibiting cell proliferation. Treating the CRC cell lines SW620 and RKO with sodium selenite significantly lowered the viability of SW620 cells at 23.1 µM (Figure 1A), but sodium selenite did not lower the viability of RKO cells, even at 28.9 µM (Figure 1B). Considering the maximum plasma sodium selenite concentration of 14.5 µM observed in clinical trials [31], sodium selenite alone might not be sufficient for effective inhibition in CRC patients, highlighting that a combinatorial compound is required to potentiate the therapeutic efficacy of sodium selenite with a clinically viable concentration. DHA, reported for its ability to inhibit the growth and proliferation of cancer cells, emerges as a promising candidate. Investigating the effect of DHA revealed that DHA concentrations ranging from 0 to 50 µM did not induce cell death in SW620 and RKO cells (Figure 1C,D). Notably, the SDCT showed significant cytotoxicity (Figure 1E,F), suggesting that DHA enhances the therapeutic effect of sodium selenite in colorectal cancer cells. SynergyFinder 2.0 [32] analysis confirmed the synergistic effect of the combination treatment, with synergy scores of 28.3 for SW620 and 23.9 for RKO (Figure 1G,H). Further validation through Calcein-AM/PI staining corroborated the synergistic effect, demonstrating significant cell death in SW620 cells treated with the combination compared to the treatments with sodium selenite or DHA alone (Figure 1I). These findings suggest that DHA significantly enhances the anticancer effect of sodium selenite.

### 3.2. DHA Remodels the Redox State within Cancer Cells

Previous studies have demonstrated that sodium selenite can enhance the generation of ROS and subsequently induce cytotoxicity in tumor cells. However, the influence of DHA on the redox state remains controversial, with some studies suggesting its antioxidant properties and others questioning its pro-oxidant effects. To investigate how DHA influences the redox state, we treated SW620 cells with DHA. 

**Figure 2:**
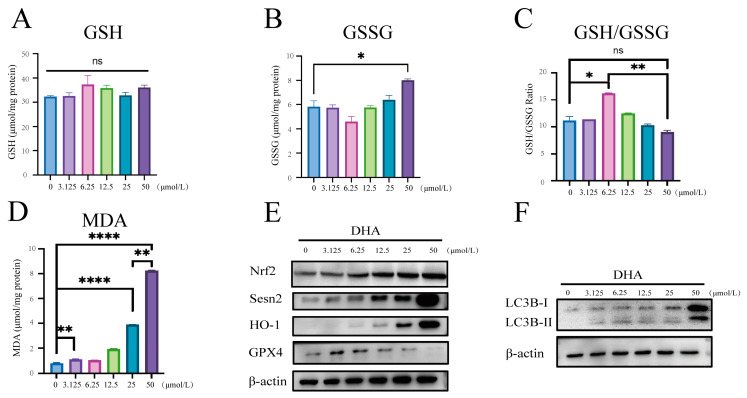
**DHA remodels the redox state within cancer cells.** (**A**–**C**) SW620 cells were exposed to DHA for 24 h, and the GSH/GSSG ratio was determined by the methods described in the Section 2. (**D**) SW620 cells were exposed to DHA for 24 h, and MDA was determined by the methods described in the Section 2. (**E**,**F**) Assessment of indicated protein levels using Western blotting in SW620 cells exposed to DHA. The corresponding quantitative histograms from three independent experiments were shown (The values represented the mean ± SD, n = 3. Two-tailed unpaired Student’s *t*-test or one-way ANOVA. ns: not significant; * *p* < 0.05; ** *p* < 0.01; **** *p* < 0.0001).

Glutathione is the most abundant non-protein antioxidant and detoxifying agent in cells and exists in reduced (GSH) or oxidized (GSSG) forms. The GSH/GSSG ratio is commonly used as an indicator of the cellular redox state [33]. Our findings revealed that the DHA treatment resulted in no significant change in GSH content, while GSSG content exhibited a trend of initial decrease followed by an increase. Consequently, the GSH/GSSG ratio was initially increased and then decreased (Figure 2A–C). Notably, at 6.25 µM, the GSH/GSSG ratio was significantly increased compared to the control, suggesting an antioxidative effect of DHA. However, at 50 µM, GSSG content was increased compared to the control, but the GSH/GSSG ratio showed no significant change, indicating that SW620 cells remained at redox homeostasis. Malondialdehyde (MDA), a final product of lipid peroxidation mediated by free radicals, reflects the level of oxidative stress within cells [34]. MDA in SW620 cells progressively increased with rising concentrations of DHA (Figure 2D), showing significant differences at all concentration points compared to the control. This indicates a continual rise in oxidative stress levels within SW620 cells. 

Despite the increase in oxidative stress levels, the cells remained in redox homeostasis. To further investigate this phenomenon, we examined the expression of antioxidant proteins. Sestrin2 (Sesn2) is a cysteine sulfinyl reductase and plays an important role in regulating antioxidative actions [35]. It has been reported that Sesn2 positively regulates the activation of Nuclear Factor Erythroid-2-Related Factor 2 (Nrf2). The Nrf2–heme oxygenase-1 (HO-1) pathway is a crucial endogenous antioxidant defense mechanism [36]. Our results showed that the Sesn2/Nrf2/HO-1 pathway was progressively activated with increasing concentrations of DHA intervention (Figure 2E), indicating that the antioxidant system within SW620 cells was gradually activated. Due to the changes in MDA and glutathione, we examined the key glutathione regulatory protein glutathione peroxidase 4 (GPX4) and found that 3.125 μM and 6.25 μM DHA interventions activated GPX4, while 12.5 μM DHA and above inhibited its activity (Figure 2E). These results suggest that as the concentration of DHA increases, cellular oxidative stress levels rise, and the pro-oxidative effect is exerted. At low concentrations of DHA, in addition to the continuous activation of the Sesn2/Nrf2/HO-1 antioxidant pathway, GPX4 and the GSH/GSSG ratio were also increased (Figure 2A–C,E), suggesting that moderate oxidative stress is beneficial for activating the antioxidant pathway and enhancing antioxidant capacity. Interestingly, despite the continued activation of the Sesn2/Nrf2/HO-1 pathway, the decrease in GPX4 and the significant increase in GSSG at high DHA concentrations suggest an overwhelming response from the GSH–GPX4 pathway. This suggests a shift in the cellular antioxidant response, potentially involving alternative pathways or compensatory mechanisms to maintain redox homeostasis.

It has been reported that autophagy induced by MDA can alleviate the cellular oxidative stress state [37]. We examined the key autophagic protein LC3B and only observed a significant increase in LC3B-II with the 50 µM treatment (Figure 2F), suggesting a pronounced activation of autophagy. This indicated that at 25 µM, SW620 cells could still rely on the enhanced activity of the Sesn2/Nrf2/HO-1 antioxidant pathway to maintain redox homeostasis, while at 50 µM, oxidative damage products further increased, necessitating the activation of autophagy to maintain redox homeostasis. Overall, our findings suggest that DHA leads to an increase in oxidative stress within cancer cells and a compensatory rise in the cellular antioxidant system. At low concentrations, it acts as an antioxidant. But at high concentrations, the pro-oxidative properties of DHA induce excessive MDA accumulation, triggering cytoprotective autophagy as a compensatory mechanism to maintain redox homeostasis and ensure cell survival. 

### 3.3. Sodium Selenite Disrupts the Redox Homeostasis Remodeled by DHA

**Figure 3:**
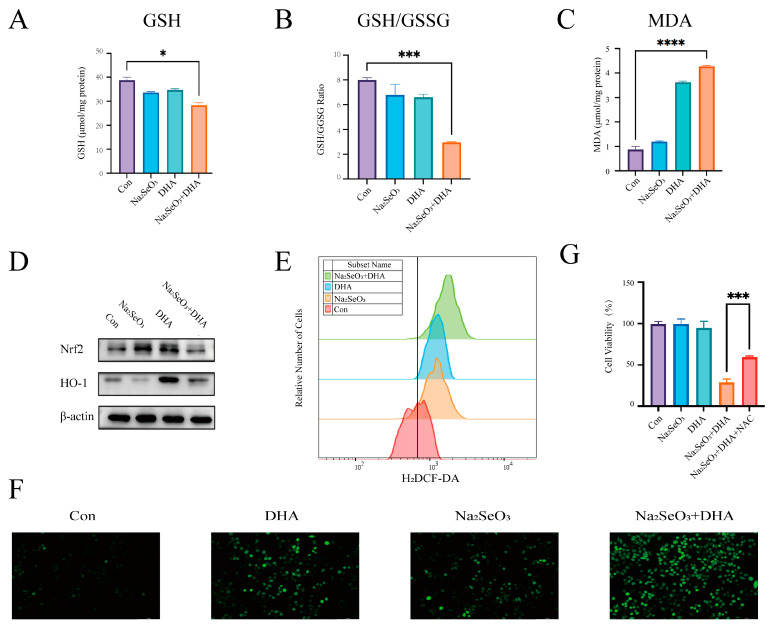
**The SDCT disrupts redox homeostasis.** (**A**,**B**) SW620 cells were exposed to 11.5 µM sodium selenite and 25 µM DHA for 24 h, and the GSH/GSSG ratio was determined. (**C**) SW620 cells were exposed to 11.5 µM sodium selenite and 25 µM DHA for 24 h, and MDA was determined. (**D**) Assessment of indicated protein levels using Western blotting in SW620 cells exposed to 1.5 µM sodium selenite and 25 µM DHA for 24 h. (**E**,**F**) SW620 cells were exposed to 11.5 µM sodium selenite and 25 µM DHA for 12 h, and the intracellular ROS level was measured by flow cytometry and a fluorescent microscope. Scale bar: 100 µm. (**G**) The viability of SW620 cells exposed to 11.5 µM sodium selenite and 25 µM DHA for 24 h with a pretreatment of 5 mM NAC (3 h). The representative images and the corresponding quantitative histograms from three independent experiments were shown (The values represent the mean ± SD, n = 3. Two- tailed unpaired Student’s *t*-test. * *p* < 0.05; *** *p* < 0.001; **** *p* < 0.0001 versus control).

To further investigate the mechanism behind the synergy between sodium selenite and DHA, we initially examined the redox state indicator. There was a significant decrease in both GSH and the GSH/GSSG ratio (Figure 3A,B), accompanied by a marked increase in MDA during the combined treatment (Figure 3C). The SDCT suppressed Nrf2 activation and reversed the DHA-induced upregulation of HO-1 (Figure 3D). Utilizing H_2_DCF to evaluate cellular ROS levels, we observed a significant enhancement in the green fluorescent signal, indicating a substantial upregulation of ROS levels in response to the SDCT (Figure 3E,F). This suggests that the combination induces oxidative stress. To verify the critical role of ROS in the SDCT-mediated cytotoxicity, we treated cells with the antioxidant NAC, which significantly reversed the cell death caused by the SDCT (Figure 3G). These findings strongly suggest that the SDCT induces ROS-dependent death in colorectal tumor cells through the disruption of redox homeostasis. 

### 3.4. The SDCT Activates the MAPK Pathway and Induces Paraptosis

**Figure 4:**
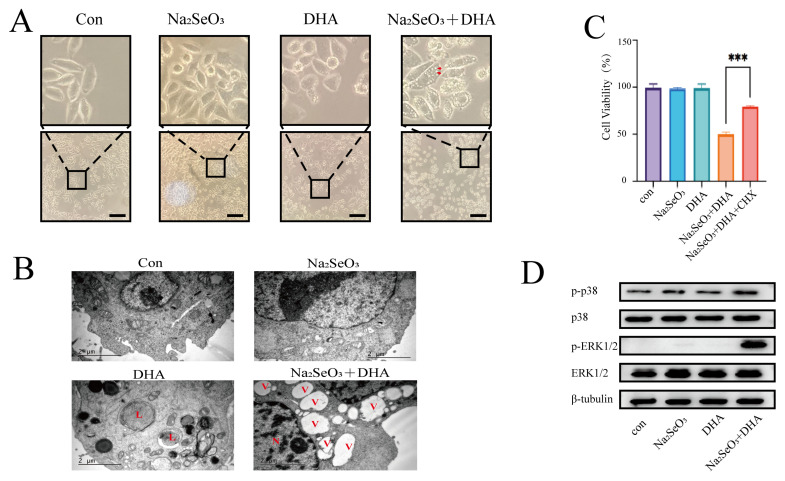
**The SDCT activates MAPKs and induces paraptosis.** (**A**) SW620 cells were exposed to 11.5 µM sodium selenite and 25 µM DHA for 24 h and observed by light microscopy. The arrows in red indicate cytoplasmic vacuoles. Scale bar: 100 µm. (**B**) SW620 cells were exposed to 11.5 µM sodium selenite and 25 µM DHA for 12 h and observed by transmission electron microscopy. The L in red indicates lysosomes. The V in red indicates cytoplasmic vacuoles. The N in red indicates a cell nucleus. Scale bar: 2 µm. (**C**) The viability of SW620 cells exposed to 11.5 µM sodium selenite and 25 µM DHA for 24 h with a pretreatment of CHX (2 h). (**D**) Assessment of indicated protein levels using Western blotting in SW620 cells exposed to 11.5 µM sodium selenite and 25 µM DHA for 24 h. The representative images and the corresponding quantitative histograms from three independent experiments were shown. (The values represented the mean ± SD, n = 3. Two-tailed unpaired Student’s *t*-test. *** *p* < 0.001 versus control).

To determine the type of cell death induced by the SDCT in SW620 cells, we examined the cell morphology and observed an accumulation of massive cytoplasmic vacuoles (Figure 4A). Further observations using a transmission electron microscope revealed that the SDCT resulted in the formation of numerous single-membrane vacuoles surrounding the nucleus, displaying characteristics of paraptosis [38]. Interestingly, transmission electron microscopy revealed lysosomal autophagic structures in the SW620 cells treated with DHA (Figure 4B), suggesting that high concentrations of DHA induce autophagy. Paraptosis is a non-apoptotic form of programmed cell death that requires protein synthesis [39]. Cycloheximide (CHX) is a protein synthesis inhibitor and has been reported to reverse paraptosis [40]. We treated SW620 cells with CHX and found that CHX reversed the cell death induced by the SDCT (Figure 4C). Mitogen-activated protein kinases (MAPKs) are a highly conserved family of serine/threonine kinases that play crucial roles in fundamental cellular processes such as growth, proliferation, death, and differentiation [41,42]. The activation of MAPKs is a distinct feature of paraptosis [43]. Our results showed that the phosphorylation of ERK1/2 and p38 were significantly elevated after being treated by the SDCT (Figure 4D), indicating the activation of MAPKs. Overall, these results suggest that the SDCT induces paraptosis in SW620 cells.

### 3.5. The Redox State Dynamically Activates the MAPKs

**Figure 5:**
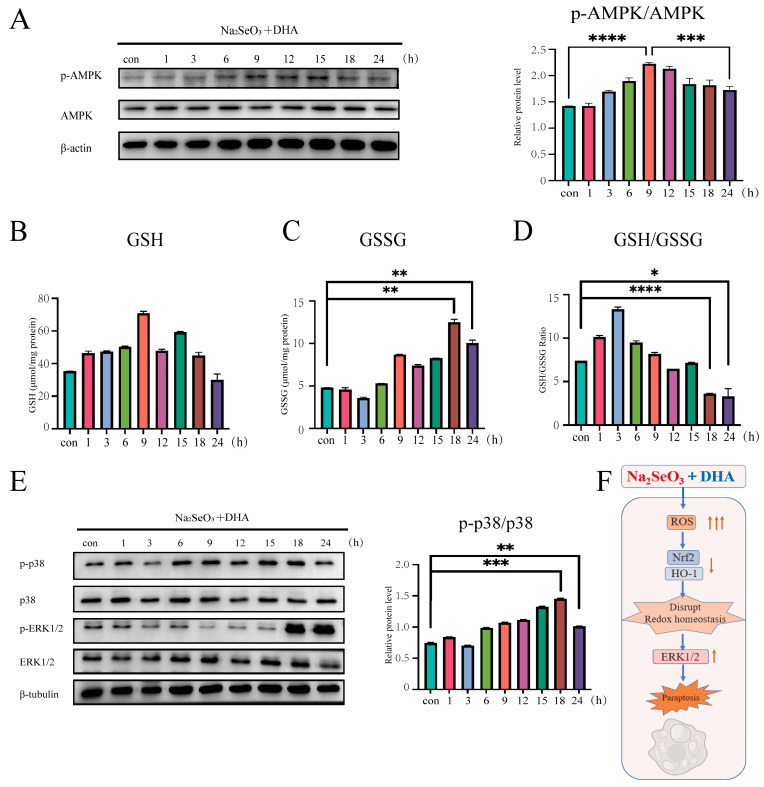
**The redox state dynamically activates the MAPK system.** (**A**) Assessment of indicated protein levels and quantitative analysis using Western blotting in SW620 cells exposed to 11.5 µM sodium selenite and 25 µM DHA. (**B**–**D**) SW620 cells were exposed to 11.5 µM sodium selenite and 25 µM DHA, and the GSH/GSSG ratio was determined by the methods described in the Section 2. (**E**) Assessment of indicated protein levels and quantitative analysis using Western blotting in SW620 cells exposed to 11.5 µM sodium selenite and 25 µM DHA. (**F**) Model of cell paraptosis induced by a combination of sodium selenite and DHA. The combined intervention produces excess ROS, inhibits Nrf2/HO-1, destroys redox homeostasis, and finally activates ERK1/2, inducing the paraptosis of CRC cells. The corresponding quantitative histograms from three independent experiments were shown (The values represented the mean ± SD, n=3. One-way ANOVA. * *p* < 0.05; ** *p* < 0.01; *** *p* < 0.001; **** *p* < 0.0001 versus the control).

Building upon our previous findings that the SDCT disrupts redox homeostasis and activates MAPKs, we further investigated the relationship between the intracellular redox state and MAPKs. Analyzing the SDCT for different durations in SW620 cells, we found that the p-AMPK/AMPK ratio, a sentinel marker reflecting the redox state [44], exhibited an initial increase, followed by a decrease, with the peak occurring at 9 h post-treatment (Figure 5A). Similarly, the GSH/GSSG ratio exhibited an initial increase, followed by a decrease, peaking at 3 h (Figure 5B–D). These initial increases suggest that SW620 cells possess a certain compensatory ability for oxidative stress. However, the significant decreases in the p-AMPK/AMPK and GSH/GSSG ratios observed at 18 and 24 h indicated the disruption of intracellular redox homeostasis. Simultaneously exploring MAPKs, we found that the phosphorylation level of ERK1/2 significantly increased at 18 and 24 h, corresponding to the decrease in GSH/GSSG ratio and p-AMPK/AMPK ratio at this time, indicating that the disruption of redox homeostasis was accompanied by the activation of ERK1/2 (Figure 5E). Interestingly, we also found that p38 was activated after treatment, while ERK1/2 was significantly activated at 18–24 h. This suggests a temporal disparity in the activation of specific proteins (p38 and ERK1/2) in the MAPK signaling pathway in response to oxidative stress.

In summary, the combination of sodium selenite and DHA inducing tumor cell paraptosis can be attributed to the disruption of redox homeostasis involving the ROS/Nrf2/HO-1 axis and hyperactive ERK1/2 signaling (Figure 5F).

## 4. Discussion

Colorectal cancer, the third most prevalent tumor globally, presents a significant therapeutic challenge due to the limited efficacy and substantial toxicity of conventional chemotherapies [1]. To address these limitations, researchers keep exploring alternative strategies, including combinatorial drug therapy, personalized treatment regimens, and nutritional modulation [45,46,47]. DHA, an omega-3 fatty acid derived from marine organisms, and selenium are both important for health and may have anticancer properties [12,24]. Despite individual reports on their anticancer activities, the potential synergistic effects of their combined administration remain unexplored. In this study, we demonstrated that DHA effectively enhanced the antitumor activity of sodium selenite in CRC cells. The SDCT significantly reduced GSH levels, the GSH/GSSG ratio, and the expression of antioxidant proteins while concurrently increasing MDA and ROS levels. Notably, the SDCT induced paraptosis-associated cytoplasmic vacuolization and activated MAPKs in the cells, suggesting a mechanism of action involving the disruption of redox homeostasis and subsequent activation of MAPKs, ultimately leading to tumor cell death. This study provides the first evidence of a synergistic anticancer effect between sodium selenite and DHA in CRC cells. This synergy holds significant potential for enhancing clinical applicability by reducing the effective concentration of each drug, thereby minimizing potential side effects and improving therapeutic outcomes.

The anticancer properties of DHA have also been revealed in recent years. However, DHA exerts its anticancer effects primarily in the non-esterified free fatty acid (NEFA) form [48,49], and this form only constitutes a small fraction (approximately 2 µM [50]) of the total plasma DHA (169 µM [51]). Previous studies have demonstrated that dietary supplementation of 1.5 g/day for six weeks can increase non-esterified DHA levels to 12.3 µM [52]. In contrast, intraperitoneal injection of ethanol-dissolved DHA directly supplements medicinal NEFA, bypassing the limitations of bioavailability. Our investigation revealed that non-esterified DHA dissolved in ethanol remodeled the redox state of tumor cells within our intervention concentration range of 0–50 µM. While it induced oxidative stress and activated the antioxidant system, it did not exhibit direct tumor inhibitory activity. Despite dietary DHA supplementation effectively raising plasma levels, even at the maximum FDA-approved daily intake of 3.0 g [53], it fails to achieve the intertumoral concentrations necessary for direct anticancer activity. Interestingly, the co-administration of sodium selenite and DHA demonstrated a synergistic tumor-inhibitory effect at a DHA concentration of 25 µM. Concomitantly, the effective concentration of sodium selenite in SW620 cells decreased to 11.5 µM, remaining below the maximum observed blood concentration of sodium selenite in clinical trials [31]. This synergistic interaction allows both agents to function at clinically achievable concentrations, offering a promising avenue for more effective clinical applications.

**Figure 6:**
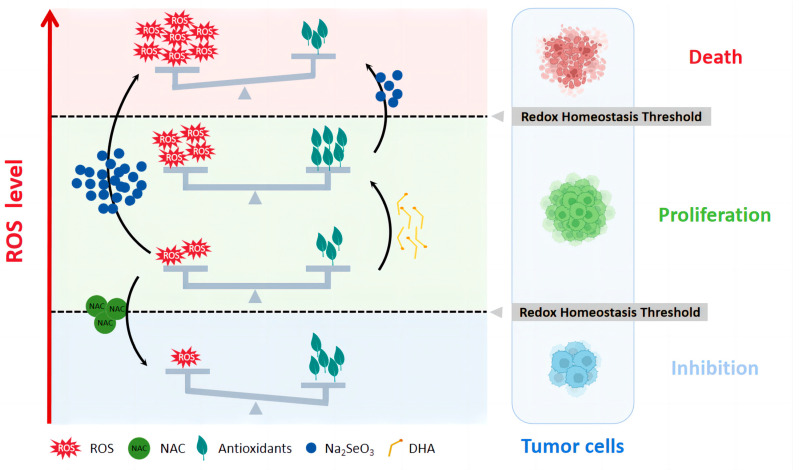
**Drug therapy based on the threshold of tumor redox homeostasis.** Tumor cells maintain redox homeostasis favorable to their proliferation, under which the cellular antioxidant is in balance with the oxidative stress level. However, there is a threshold for homeostasis. When the oxidative stress is higher than the limit of cellular antioxidant compensation or lower than the concentration necessary to maintain tumor proliferation, tumor cells will be inhibited from proliferating and eventually die. Reducing oxidative stress by using antioxidants, such as NAC, inhibits tumor cell growth by impairing redox homeostasis. DHA increases oxidative and reductive levels in tumor cells but remains within the redox homeostatic range. The combination of low concentrations of sodium selenite with DHA induces the production of ROS above the redox homeostasis threshold, which would be achieved with high concentrations of sodium selenite alone. By co-regulating the redox state, sodium selenite and DHA synergistically induced tumor cell death.

ROS, as metabolic byproducts, play a significant role in regulating tumor development and progression [54]. Tumor cells exhibit an abnormal redox state with excessive proliferation and high levels of ROS generation [55,56]. Correspondingly, tumor cells enhance their antioxidant capacity by activating the Nrf2 pathway [57] and elevating cellular GSH levels to avoid cellular senescence, apoptosis, or ferroptosis induced by excessive ROS so as to allow tumor cells to adapt to and depend on high oxidative stress [36,58]. Nevertheless, this adaptation to ROS is constrained by the redox homeostasis threshold(Figure 6). When oxidative stress exceeds the limit of cellular antioxidant compensation or falls below the concentration required to sustain tumor proliferation, cell proliferation will be inhibited and eventually lead to cell death. Our previous research showed that reducing ROS levels in tumor cells using NAC causes them to fall below the threshold of redox homeostasis [59]. This leads to impaired redox homeostasis, an inhibition of cell growth, and eventually the death of tumor cells. Therapeutic strategies aimed at disrupting redox homeostasis by increasing ROS levels hold promise for treating cancer, particularly in tumor cells with high ROS and antioxidant capacity susceptible to surpassing the threshold. Our research results indicate that physiological doses of DHA can simultaneously increase oxidative and antioxidative stress, making CRC cells more susceptible to oxidative stress. When combined with low doses of sodium selenite, this leads to elevated ROS levels that exceed the redox homeostasis threshold, ultimately resulting in cell death. 

Tumor cells display increased sensitivity to oxidative stress as compared to normal cells, suggesting that this vulnerability can be exploited by a therapeutic strategy of inducing oxidative stress to target and inhibit tumor growth [27,28]. DHA has been shown to mitigate oxidative stress by upregulating HO-1 [60], an antioxidant enzyme overexpressed in tumors that contributes to chemoresistance [61,62]. We propose that within the threshold of redox homeostasis, chemotherapeutic drugs elevate ROS levels, thereby enhancing the expression of Nrf2 and HO-1, sustaining an elevated redox balance. Our data also confirmed that interventions with either sodium selenite or DHA alone lead to an upregulation of Nrf2, supporting our hypothesis. However, when ROS levels surpass the redox homeostasis threshold, there was a decline in Nrf2 and HO-1 expression, indicating a disruption of redox equilibrium. This fundamental principle underpins the rationale for the combined intervention of sodium selenite and DHA. By leveraging the ability of DHA to remodel the redox states within the tumor microenvironment, a small dose of sodium selenite can achieve comparable effects to those typically induced by high levels of ROS. This targeted approach disrupts the delicate redox homeostasis within tumor cells, ultimately leading to their death. 

We found that the SDCT induced paraptosis after disrupting redox homeostasis. The mechanism of this process involves complex interactions between ROS regulation and MAPK signaling. P38 plays a pivotal role in the tumor’s response to external stimuli as a stress-activated protein kinase, including oxidative stress, leading to tumor cell apoptosis [63]. Our results, consistent with previous reports, suggested that ROS can activate P38 [64]. The relationship between ERK1/2 and tumor growth remains unclear. While ERK1/2 activation has been associated with tumor cell proliferation [65,66], recent studies have shown that the overactivated ERK1/2 can paradoxically promote paraptosis [43]. Additionally, the relationship between ROS and ERK1/2 is complex. ROS can promote cell proliferation and survival by activating ERK1/2 [67,68], but there are also reports suggesting that ROS can inhibit ERK1/2, leading to tumor cell apoptosis and autophagy [69]. Our results revealed that ERK1/2 levels initially decrease and then increase with increasing oxidative stress, suggesting a close link between ERK1/2 activity and the tumor cell redox state. Within the redox homeostasis threshold, increased ROS levels suppress ERK1/2. However, when ROS levels exceed the threshold, ERK1/2 is overactivated, leading to paraptosis. These findings highlight the dose-dependent nature of ROS-induced ERK1/2 activation, providing valuable insights into the complex relationship between ROS and ERK1/2 signaling. This knowledge can potentially guide the development of more effective therapeutic strategies for cancer treatment.

While increasing oxidative stress can inhibit tumor cells, excessive oxidative stress is detrimental to normal cells. This highlights the critical need to determine the appropriate range of oxidative stress for effective and safe tumor treatment. This challenge necessitates further exploration of reliable biomarkers of redox homeostasis, which can provide valuable insights into the redox state of tumors and guide the precise administration of therapeutic interventions. Our study employed P-AMPK/AMPK and GSH/GSSG, previously reported markers of redox homeostasis [44]. However, these biomarkers have certain limitations, prompting the need to identify additional markers that more accurately reflect the redox state of tumors. This will enable us to precisely determine the threshold of redox homeostasis in individual tumors, thereby guiding the development of personalized treatment strategies that maximize efficacy while also minimizing the adverse effects associated with high-dose chemotherapeutic drugs.

This study paves the way for clinical applications and future research. Our findings demonstrated that DHA could remodel the redox state within cancer cells, making them more susceptible to oxidative stress and thereby increasing their sensitivity to sodium selenite. These data provide a preliminary basis for clinical research on the beneficial effects of dietary intakes of DHA. Given that dietary fatty acids are absorbed in the intestine, leading to higher local concentrations, and that intestinal tumor cells exhibit a higher uptake of fatty acids compared to normal cells [70,71], DHA is more likely to accumulate in CRC cells than in normal cells [72]. This selective accumulation allows DHA and chemotherapeutic drugs to act synergistically in tumor cells without affecting normal cells, offering a safer and more targeted treatment strategy for CRC patients, especially those requiring adjuvant chemotherapy. This finding warrants further investigations in preclinical models and clinical trials to assess the efficacy and safety of this combination therapy in clinical settings. Concurrent dietary DHA management during treatment could not only enhance therapeutic outcomes but also mitigate the progression of cachexia to some extent, showing strong clinical translational potential. Future research should explore the synergistic effects of polyunsaturated fatty acids with other chemotherapeutic or targeted drugs to broaden their clinical application. Furthermore, our hypothesis of focusing on redox homeostasis offers a new approach for more specific and less toxic cancer treatments, thus expanding the horizons of tumor therapy.

## 5. Conclusions

In conclusion, this study provides a compelling case for the use of sodium selenite and DHA as a combinatorial treatment strategy for CRC. By enhancing our understanding of redox homeostasis in cancer cells, this research paves the way for developing more effective and less toxic therapeutic interventions.

## Data Availability

Data are contained within the article.
